# Exposure to Inorganic Nanoparticles: Routes of Entry, Immune Response, Biodistribution and In Vitro/In Vivo Toxicity Evaluation

**DOI:** 10.3390/toxics5040029

**Published:** 2017-10-17

**Authors:** Valeria De Matteis

**Affiliations:** Dipartimento di Matematica e Fisica “Ennio De Giorgi”, Università del Salento, Via Arnesano, 73100 Lecce, Italy; valeria.dematteis@unisalento.it

**Keywords:** inorganic nanoparticles, in vitro/in vivo toxicity, routes of entry, immune response, biodistribution

## Abstract

The development of different kinds of nanoparticles, showing different physico-chemical properties, has fostered their large use in many fields, including medicine. As a consequence, inorganic nanoparticles (e.g., metals or semiconductors), have raised issues about their potential toxicity. The scientific community is investigating the toxicity mechanisms of these materials, in vitro and in vivo, in order to provide accurate references concerning their use. This review will give the readers a thorough exploration on the entry mechanisms of inorganic nanoparticles in the human body, such as titanium dioxide nanoparticles (TiO_2_NPs), silicon dioxide nanoparticles (SiO_2_NPs), zinc oxide nanoparticles (ZnONPs), silver nanoparticles (AgNPs), gold nanoparticles (AuNPs) and quantum dots (QDsNPs). In addition, biodistribution, the current trends and novelties of in vitro and in vivo toxicology studies will be discussed, with a particular focus on immune response.

## 1. Introduction

A “nanomaterial” is defined as a “material with any external dimension in the nanoscale or having internal structure or surface structure in the nanoscale” by the International Organisation for Standardisation (ISO, 2010) [1]. On the other hand, “nanoparticles (NPs)” are “nano-objects with all three external dimensions in the nanoscale”, where the nanoscale was defined as the size ranging from 1 nm to 100 nm (ISO, 2008) [2]. Several natural processes, such as volcanic eruptions, dust storms, and human industrial activities produce NPs. As a consequence, living organisms are continuously exposed to NPs, which access the human body through different routes [3]. At the same time, human industrial activities have contributed to the wide spread of NPs, especially those of few nanometers [4]. In addition, the variability of chemical composition increases the dangerousness of NPs. This has caused a rise in respiratory, cardiovascular and tumor diseases related to high concentrations of NPs in the atmosphere [5]. Particle fractions smaller than 2.5 µm (PM2.5) and ultrafine particles (UFPs) with a size of 0.1 µm are demonstrated to affect human health much more than microsized NPs (PM10) with a diameter of 10 µm [6]. Different sources of atmospheric UFP emission in the UK were identified by the report of the Airborne Particles Expert Group in 2005 [7]: road transport produced the major source of NPs (60%), whereas 23% derived from industrial and residential combustion processes [7]. The term “engineered nanoparticles” (ENPs) was introduced to distinguish natural from manufactured particles sharing similar size [8]. The ENPs are produced by different chemical synthetic methods: the bottom-up or top-down approaches [9]. Based on their composition, ENPs are classified in three main groups. The first is characterized by inorganic NPs that include quantum dots (QDs), metal oxide NPs (MONPs) such as SiO_2_NPs, TiO_2_NPs, ZnONPs and metallic NPs such as silver NPs (AgNPs) and gold NPs (AuNPs). The second group includes organic nanoparticles, synthetized with organic compounds such as chitosan nanoparticles. The third group includes carbon-based nanoparticles such as carbon nanofibers and carbons nanotubes (CNTs) [10]. The different physico-chemical properties of ENPs are demonstrated to drive adverse effects in biological systems [11]. For example, it is recognized that a small dimension induces more toxic consequences than larger nano-objects due to greater surface area per mass unit [12]. The properties of ENPs can be modified by functionalization processes, in order to change or reduce the toxicity of the chemical materials used for their production [13]. In addition to the physico-chemical properties, the interaction between NPs and the culture medium, or biological fluids, is another crucial parameter to take into consideration in toxicology studies [14]. This interaction could promote NP aggregation and dispersion, both influencing cell uptake with a consequent impact on the NPs toxicity [15]. To this respect, Wartheit et al. [16] and Shvedova et al. [17] have underlined the inaccuracy of evaluating NP toxicity, both in vitro and in vivo, using agglomerated and unstable NP suspensions. For this reason, several investigations focused first on the preparation of stable NPs dispersion [18,19]. It is widely accepted that aggregation is strongly dependent on different factors, such as surface coatings/charge, materials, solution properties, etc. Then, it is quite difficult to predict NPs stability in media [20]. Today, the adverse effects of NPs are still unclear; deeper investigations of toxicity mechanisms in living organisms are required [21]. The trigger of reactive oxygen species (ROS) causes an oxidative stress cascade, responsible for several diseases, including cancer [22]. The growing use of commercial products, based on ENPs, requires special care regarding the risk assessment, aiming at synthetizing safer nanomaterials [23]. For this reason, nanotoxicology is a recent discipline that aims at identifying the relationship between exposure route and dosage, and to assess the role played by the NPs physico-chemical properties [24]. Nanotoxicology is orientated to gathering and evaluating data obtained from in vitro and in vivo studies, in order to increase useful knowledge regarding the use of nanomaterials and strategies to protect living organisms [25]. In general, in vitro investigations are a very useful to enrich clinical and epidemiological studies, a topic particularly relevant for companies producing NM-based commercial products [26]. Chronic toxicity investigation and also biodistribution and toxicokinetics in organs require in vivo assessment [27]. Computational methods are also important tools in the nanotoxicology field, and allow overcoming in vivo studies that have both ethical limitations and long experimental times [28]. In silico toxicology can be useful to predict the toxicity of NMs; this tool is essential for reinforcing the standard in vitro tests and, in some cases, to design a specifically safe nanocarrier for drug delivery applications [29]. In this review, the entry route of inorganic NPs (SiO_2_NPs, TiO_2_NPs, ZnONPs, AgNPs, AuNPs, QDsNPs), the immune response and the toxicity assessment are investigated taking into account in vitro and in vivo studies.

## 2. Regulation of Nanomaterials Risk Assessment 

The current evaluation methods for the regulation of nanomaterials are inadequate to establish and to foresee the risks to health. The USA and Europe have developed many approaches for nanomaterial regulation. The Environmental Protection Agency (EPA) regulates the use of NPs in the USA, in collaboration with other federal government agencies, such as the Food and Drug Administration (FDA). Chemical management is ruled by the ‘Registration, Evaluation, Authorization and Restriction of Chemicals’ (REACH) regulation in Europe. Therefore, there is not an adequate differentiation between nanomaterials and chemical substances [30]. Moreover, the European member states, in collaboration with the Chemicals Agency (ECHA), published a series of documents regarding the use of nanomaterials in industries, enriching the registration of nanomaterials in REACH [31].

## 3. NPs Routes of Entry and Toxicity

The main routes of entry of inorganic NPs (ingestion, inhalation, dermal penetration, blood circulation) and toxicity assessment by in vitro and in vivo studies are summarized in Table 1. This section highlights the importance of the physico-chemical properties and the dosage as relevant parameters for establishing a NPs toxicity profile. SiO_2_NPs are used in the food industry, powders, and in health care products such as toothpastes, detergents, and cosmetics [32]. TiO_2_NPs and ZnONPs are employed in commercial products such as sunscreens, food additives, and paints [33]. Inevitably, ingestion and skin penetration are the principal, although not the exclusive, way to entry into the human body. AgNPs are used in food, in antibacterial products, water disinfectants, textile industries, diagnostic biosensors, imaging probes and conductive inks [34]. For these reasons, they can access the human body by inhalation, ingestion and skin penetration. AuNPs and QDs find their major diffusion in the medicine field; phototermal therapy, bioimaging and drug delivery are the principal applications [35,36].

### 3.1. Ingestion

**Figure 1:**
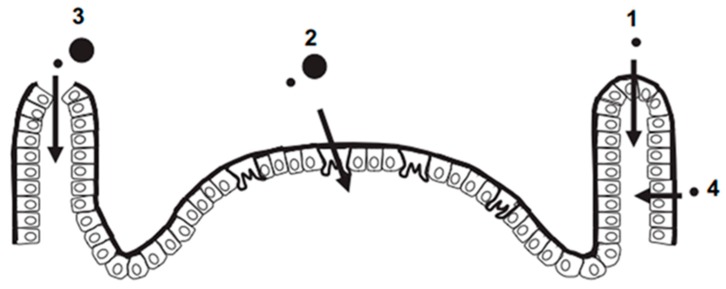
Particles crossing the gastrointestinal tract. 1. Small NPs (<50–100 nm) cross the ‘regular’ epithelial cells by endocytosis. 2. Large NPs and small microparticles cross the M-cell by transcytosis. 3. Feasible passage through villous ‘gaps’ of nano and microparticles. 4. Paracellular uptake of small NPs. Reprinted from reference [89]. Copyright (2010), with permission from Elsevier.

The human gastrointestinal apparatus has a large surface area (200 m^2^), which is divided into the upper (esophagus, stomach, and duodenum) and lower gastrointestinal tract (small intestine and all of the large intestine), each one having specific functions [79]. This apparatus represents a mucosal barrier that selectively promotes the degradation and uptake of nutrients such as carbohydrates, peptides, and fats. Humans can ingest directly a lot of food ingredients, additives and supplements containing NPs [80]. A huge number of inhaled nanoparticles can move through the trachea with the help of mucociliary cells, and indeed entering the stomach [81]. NPs can act on the motility of the gastrointestinal system, on the mucus layer covering the lumen and on the microbiota [82]. All of these factors are necessary to maintain the physiological homeostasis of the body and the immune system [83]. However, NPs can translocate into the bloodstream and consequently access each organ upon crossing the epithelium [84]. The translocation of NPs into and across the gastrointestinal mucosa can occur by different routes. In the gastrointestinal tract, there is a modulation of pH value, changing along the various sections of the apparatus [85]. In addition to pH, peristalsis can affect NP physicochemical properties, as the pressure can reach 150 mm Hg [86]. The stomach is characterized by an acidic environment with an early-stage pH range of 1.2–2.0. When the bolus is formed, however, the pH reaches values of c.a. 5.0, followed by slow re-acidification [87]. The dissolution of NPs at a low pH promotes their further degradation in the digestive fluids. Degradation phenomena and ion release were observed in gastric fluids, even though the NP stability *in loco* has not been clarified. It has been observed that the uptake of NPs with a diameter lower than 100 nm occurs mainly by endocytosis [88] in “regular” epithelial cells. Digestion and absorption processes are carried out by the small intestine that, at the duodenum site, has a typical pH value between six and seven [89]. The enhancement of the absorptive surface is due to the presence of villi and enterocytes. In the intestinal cells, NPs can trigger oxidative stress, DNA damage and inflammations [90]. Large NPs and microparticles can also cross the intestinal epithelium through transcytosis by M-cell-uptake and persoption. Moreover, NPs can cross the villi through the gaps formed in their apical zone due to a dysfunction process induced by NPs altering the morphology of the epithelium [91]. NP paracellular uptake is a rare process but, in disease conditions, the junctions of cells can undergo an alteration promoting the passage of NMs [89] (Figure 1).

**Figure 2:**
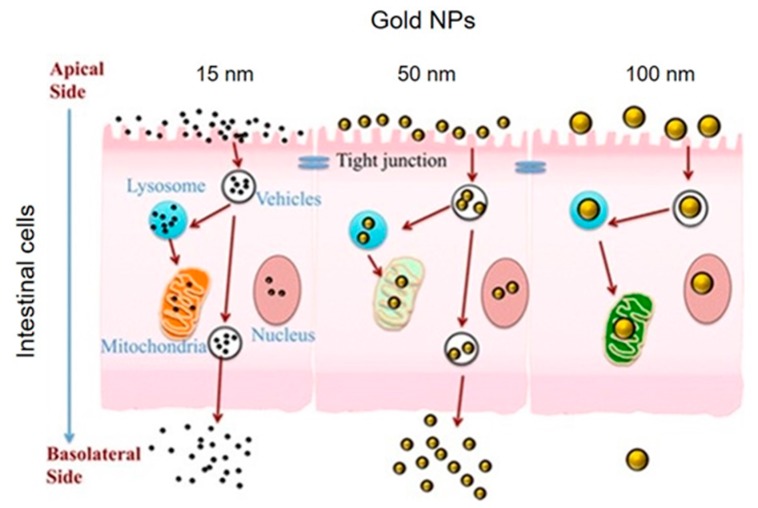
Relationship between AuNP size and uptake across intestinal cells. AuNPs of 15 nm were absorbed by the intestinal epithelial and spread quickly throughout and into cells. AuNPs of 50 nm crossed the apical side of the intestinal epithelium and were excreted via the basolateral side. AuNPs of 100 nm accumulated in intestinal cells and consequently the excretion route is very low. Reprinted (adapted) with permission from reference [46]. Copyright (2015), American Chemical Society.

In general, the gastrointestinal tract undergoes the exposure of ‘exogenous’ and ‘endogenous’ NPs [92,93]. TiO_2_ or silicates/aluminosilicates are classified as exogenous inorganic particles [94] and they are largely used in food industries and in the pharmaceutical industry as an additive [95]. The commercial labelled E171 for TiO_2_ is also present in sweets [96] with the quantity ranging between 1–5 µg/mg of E171. In this case, the authors estimated a daily ingestion of around 31.5 mg/day for a 70 kg person [97]. Since 2007, NP-based products have been dramatically growing in food industries. AgNPs, TiO_2_NPs, ZnONPs, SiO_2_NPs are the most commonly used [98]. The daily consumption of AgNPs by ingestion is estimated around 20–80 µg [99]. E551, E554, E556, or E559 are commercial labels of SiO_2_NPs used as food additives and their ingestion was estimated around 126 mg/day for a 70 kg person [100]. This is rather important, as the limits accepted by the Scientific Committee on Food of the European Food Safety Authority is 20–50 mg SiO_2_NPs for a 60 kg person [101]. Intestinal cells incubated with TiO_2_, SiO_2_, Ag and ZnONPs have shown an induction of cytokine secretion [37,38]. TiO_2_NPs and SiO_2_NPs showed lower genotoxicity with respect to AgNPs at the same size [39]. Furthermore, the targets of the toxic effects of NPs are the cellular junctions and microvilli of enterocytes. In a recent study, Caco-2 cells (caucasian colon adenocarcinoma) exposed to 10 μg/mL TiO_2_NPs showed a disruption of microvilli and tight junctions [40]. The TiO_2_ extracted from chewing-gums induced collapse of the enteral epithelium [85]. SiO_2_NPs with a size of 70 nm, 300 nm and 1000 nm surface modified with carboxyl or amine groups were used to study the oral exposure in BALB/c mice (female, six weeks) with a concentration of 2.5 mg/mouse for 28 days every day. The adsorption by intestinal cells was significantly relevant for SiO_2_NPs with a size of 70 nm functionalized with carboxyl groups, suggesting the important role of surface in the uptake. The authors, in this case, did not find relevant toxicity in mice [48]. Wei et al. showed cytotoxicity events in the human colorectal carcinoma cell line (HCT 116). In particular, the exposure to low (10 μg/mL) or high dosages (200 μg/mL) of NPs induced the transformation of microtubule-associated protein 1-light chain 3-I (LC3-I) to LC3 (LC3-II) after 24 and 48 h of incubation. Moreover, the resulting autophagy was only time dependent, but not dose dependent [41]. On the other hand, Sergent et al. confirmed low cytotoxicity measured as global metabolism activity of human epithelial intestine cells HT-29 exposed to SiO_2_NPS (25 and 110 nm) for 24 h [42]. The deposition of AgNPs in the gastrointestinal tract has been widely demonstrated in previous studies [49,50]. In addition, AgNPs (5–20 nm) orally administrated for 21 days in mice (20 mg/kg of BW) eventually disrupted epithelial cell microvilli and intestinal glands [51]. Moreover, Jeong et al. showed an increase of goblet cells (columnar epithelial cell) in the intestine, together with high mucus granule release in mice treated with AgNPs (60 nm), with a concentration of 30 mg/kg of bw/day for 28 days [52]. Böhmert et al. analyzed the toxicity of AgNPs with a primary size of 7.02 ± 0.68 nm in Caco-2 cells using NP concentrations between 1 and 100 μg/mL. Partial aggregation between digested and not-digested particles was observed by field fractionation (A4F) combined with dynamic light diffusion (DLS) and X-ray dispersion at small angles (SAXS). The authors concluded that AgNPs entered the gastrointestinal barrier without forming large aggregates in digestive fluids. These results confirmed the importance of body fluids on NP behavior and toxicity [43]. Tada-Oikawa et al. investigated the effects of TiO_2_NPs rutile and anatase (50, 100, 250 nm) in Caco-2 cells and THP-1 monocyte-derived macrophages in a range of concentrations between 1 to 50 µg/mL for 24 or 72 h. Results showed a dose-dependent reduction of cell viability together with a high amount of ROS generation. A total of 50 µg/mL of anatase (50 nm) TiO_2_NPs induced the production of interleukin (IL)-1β in THP-1 macrophages. On the other hand, they induced an increase of IL-8 expression in Caco-2 cells [44]. Koeneman et al. showed the inability of TiO_2_NPs to distrupt γ-catenin proteins in Caco-2 cells exposed to a concentration of 10 μg/mL of NPs. Moreover, TiO_2_NPs induced an alteration of microvilli on the apical surface of the epithelium and an increase of calcium level [45]. Yao et al. suggested that the absorption rate of AuNPs increases with size—100, 50 and 15 nm (5 µg/mL)—by Caco-2 cells. NP uptake was more evident for AuNPs of small size, but the authors observed a decrease of accumulation in cells that triggered a depolarization of mitochondria membranes causing cytotoxicity [46] (Figure 2). Moos et al. assessed the toxicity of nano (8–10 nm) and micro (<44 μm) ZnONPs on human colon carcinoma cells (RKO), observing a mithocondrial dysfunction. The adverse effects were correlated to the interactions between the particles and cell; the toxicity did not depend to zinc ions in cell culture medium. The NPs were observed to be more toxic than microparticles. The LC_50_ value was 15 ± 1 for smaller and 29 ± 4 μg/cm^2^ for larger NPs [102]. Wang et al. showed the toxicity of CdSe quantum dots (QDs) with PEG shell on Caco-2 cells, and the effect of artificial gastric fluid on CdSeQDs toxicity after 24 h. The acidic conditions of gastric fluids (pH 1.2) induced a disruption of the PEG shell, uncovering the CdSe core, resulting in strong cell mortality [47].

In all of the reported works, the influence of the size and physico-chemical properties of the toxicity evaluation in the gastrointestinal apparatus is evident. Nevertheless, the QDs and AgNPs are less tolerated even at low concentrations, due to their susceptibility to low pH, which induces a degradation process.

### 3.2. Inhalation

**Figure 3:**
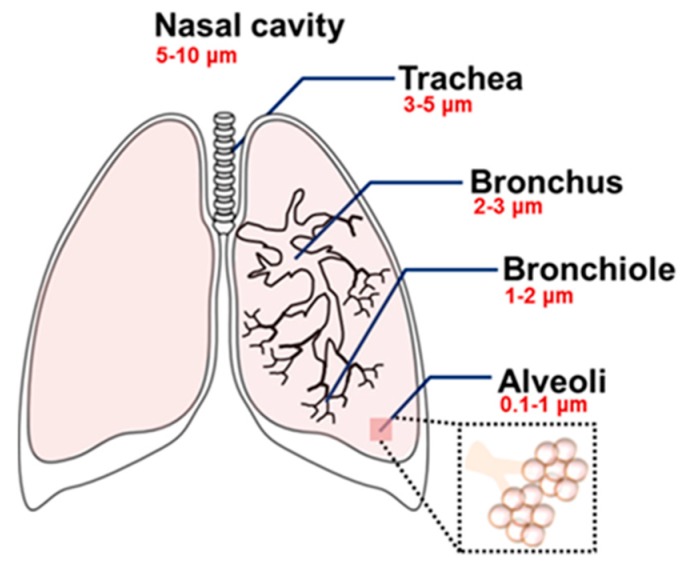
Different airway regions host NPs, showing the size-dependent deposition of NPs. Microsized particles arrest the upper airway regions, while smaller particles reach the deepest lung region (alveoli). Reproduced with permission from reference [114].

The respiratory system consists of two parts: the upper respiratory tract, which includes the nasal cavity, the pharynx, and the larynx, and the lower respiratory tract, which includes the trachea, the bronchi and the lungs. Each bronchus is branched in small structures called bronchioles that are connected with alveoli, responsible for gas exchange [103,104]. Wibel et al. described the structure of the respiratory system that is composed of 23 bifurcated tubes numbered from G0 to G23. Among these, the airways from G17 to G23 have a gas exchange function. The upper respiratory tract not only allows air passage, but also protects the lower respiratory tract. Despite this, NPs are able to enter the respiratory system [105]. The size distribution of NPs plays an important role in their ability to enter the human respiratory tract [106]. Larger NPs, with a diameter between 5 and 30 μm, usually remain in the nasopharyngeal region, whereas smaller NPs, with a size between 1 and 5 μm, tend to deposit in the tracheobronchial region. In addition, small NPs (0.1–1 µm) can reach the alveolar region, which is the deepest region of the respiratory system, by gravitational sedimentation and Brownian diffusion [107,108]. Once the NPs are deposited, the elimination mechanisms are not fast and many toxics effects can be generated because of the prolonged interaction with cells [53]. Body mechanisms such as mucociary and coughing can promote NP motion, except for smaller NPs that reach the deepest alveolar region. For these NPs, the expulsion is insufficient [109,110] compared to larger NPs [111]. In addition, translocation in blood capillaries is very easy for particles with a diameter lower than 0.5 μm [112] (Figure 3). The epithelium, consisting of a monolayer of epithelial cells (type I and type II), that divides the inhaled air from blood capillaries is very thin [113]. Type I and type II cells maintain the homeostasis of lungs. When damage occurs, a penetration of NPs can be observed.

Pujalté et al. exposed male Sprague-Dawley rats to 15 mg/m^3^ of anatase TiO_2_NPs (~20 nm) for 6 h via the inhalation route. After the sacrifice of the rats, they found high levels of Ti in the lungs, but also in the liver, kidney, and spleen. In addition, the TiO_2_NPs were mainly eliminated by faeces and urine, suggesting that the NPs elimination process occurs by mucociliary clearance and ingestion [62]. Miller et al. demonstrated that inhaled AuNPs can reach the blood circulation, accumulating at sites of vascular inflammation. This was demonstrated in animal models and in men. In particular, fourteen healthy male volunteers were exposed to inhalation of AuNPs with size of 3.8 nm or 34 nm (116 ± 12 μg/m^3^; 5.8 ± 0.3 × 10^6^ NPs/cm^3^ for 2 h. This evidence suggests that the fate of inhaled nanoparticles, as well as their size, is crucial for their potential to induce cardiovascular disease. These phenomena were size dependent [63]. Citrate-capped AuNPs (13 nm) indicate high toxicity in lung carcinoma cells, while no adverse effects were observed in liver carcinoma cells [54]. In a recent work, the toxicy of inhaled ZnO < 50 nm was investigated in an in vitro air-blood barrier (ABB) model, consisting of transwell co-cultures of lung epithelial cells (NCI-H441) and immortalized pulmonary microvascular endothelial cells (HPMEC-ST1 6R). The authors observed the release of pro-inflammatory mediators (IL-6, IL-8), followed by the secretion of dysfunction markers (sICAM-1 and sVCAM-1). In particular, 260 pg/mL of IL-8 were detected in treated cells versus control cells [55]. Exposure of primary human bronchial epithelial cells (BEAS-2B) to ZnONPs triggered cytotoxicity, oxidative stress, high intracellular Ca^2+^ levels and alteration of mitochondrial membrane potential. The same effects were observed on adenocarcinomic human alveolar basal epithelial cells (A549) [56]. Xu et al. confirmed the onset of lung tumors, broncho-alveolar adenomas and cystic keratinizing squamous cell carcinomas after the inhalation and instillation of rutile and anatase TiO_2_NPs [64]. Lee et al. exposed A549 cells to increasing concentrations of AgNPs for 24 h, and found morphological changes and cell death in a dose-dependent manner [57]. The dose-dependent toxicity of uncoated (20, 40, 60 and 80 nm) and PVP-coated AgNPs (10, 50, and 75 nm) was investigated in a macrophage cell line (J774A.1) by Nguyen et al. [58]. The results clearly showed a cell shrinkage effect due to uncoated AgNPs, whereas cell elongation was evident after treatment with PVP-coated AgNPs. These evidences suggested that the coating induced different cell effects. Prolonged inhalational exposure to AgNPs induced lung function alterations and inflammatory responses [65]. AgNPs accumulation was more evident in the lungs and liver after 90 days of inhalation [66]. AgNPs (20 nm) induced DNA damage and overexpression of metallothioneins in A549 cells at a concentration of 0.6 nM up to 48 h [59]. The biodistribution and toxicity are dependent on the size, shape and surface properties. Small AgNPs were accumulated in different organs whereas the larger ones preferred the liver and spleen. In addition, the AgNPs induced adverse effects in a dose-dependent manner (the concentrations tested were 4, 10, 20, 40 mg/kg) [67]. The in vitro effects of 50 μg/mL of SiO_2_NPs (10, 150, and 500 nm) were investigated in human lung submucosal cells (Calu-3) for 2–24 h. NPs induced high levels of malondialdehyde (MDA) and genotoxicy [60]. Lin et al. investigated toxicity in a human bronchoalveolar carcinoma-derived cell line (A549) exposed to SiO_2_NPs (15 nm, 46 nm) at 10, 50, and 100 μg/mL for 48 h, showing that SiO_2_NPs induce ROS production, lipid peroxidation and loss of viability [61].

All this evidence suggests that size is the principal physico-chemical property with impacts on the airways. On the other hand, smaller NPs are more able to spread and accumulate in the deepest regions, thus triggering inflammations and ROS production.

### 3.3. Skin Penetration

**Figure 4:**
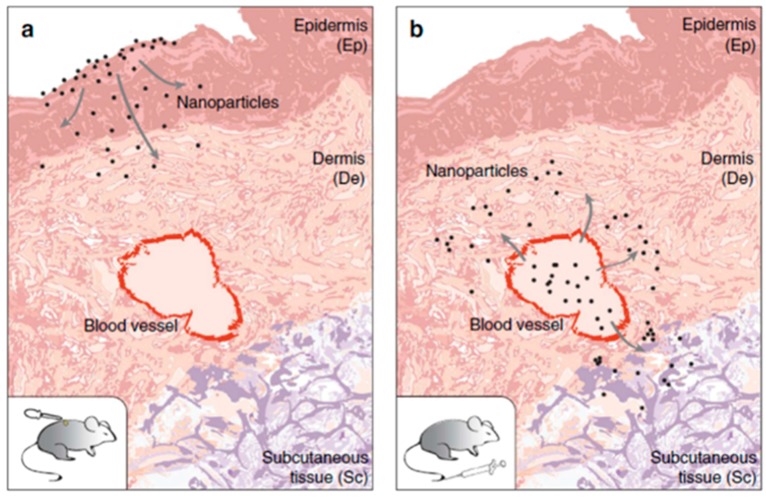
Route of entry of NPs in mice after topic dermal application (**a**) and tail-vein injection (**b**). When NPs were applied topically, they spread in the epidermis and dermis. The subcutaneus tissue (hypodermis) was reached by NPs only after the injection. Reprinted by permission from Macmillan Publishers Ltd. Nature Communication copyright (2014) [78].

The skin is a dynamic organ that has different functions, such as protection against external agents, UV protection and a selective permeable barrier. The skin also plays a crucial role in the regulation of human body temperature and immunological response, due to the presence of Langerhans cells. The skin is structurally divided into three layers: epidermis, dermis and hypodermis [115]. With the development of nanomaterials, their application is growing in the cosmetics field. Since 2006, NP-containing cosmetics have shown a large diffusion [116]. It has been estimated that cosmetic products contain TiO_2_NPs (70/80%), ZnONPs (70%) and AgNPs (20%) [77,117]. Some studies observed the impossibility of NPs crossing the skin barrier. In contrast, other studies showed the penetration of metal NPs, such as iron NPs, through hair follicles [118], reaching the basal and spinous layers [119]. Also in this case, the entry of NPs through the skin is influenced by size. The available data shows that NPs with a diameter of around 4 nm can penetrate intact skin, whereas has been observed that with an increase in size up to 45 nm, NPs can only permeate damaged skin [120]. Natural and artificial radiation can induce a disaggregation of TiO_2_NPs, as few minutes of irradiation leads TiO_2_ aggregates to decrease from 280 nm to 230 nm. Pig skin was used to evaluate the penetration of TiO_2_NPs in light and dark conditions. In the presence of light, 200 mg/kg^−1^ was found in the skin, whereas in dark conditions only 75 mg kg^−1^ was found, suggesting that photoinduction can promote NP dermal penetration [68]. The same phenomenon was described by De Matteis et al., who founds a disaggregation not only in the presence of light, but also due to pH skin action (5.5). In addition, rutile TiO_2_NPs showed lower toxicity than the anatase form [69]. Crosera et al. evaluated the toxicity of TiO_2_NPs on human epidermal keratinocyte (HaCaT cells) for 24, 48 h and seven days, observing low cytotoxicity. The results demonstrated the potential toxic effects only after exposure to high concentrations (0.007–50 μg/cm^2^) up to seven days [70]. ZnONPs induce a disruption of the mitochondria in HaCaT, and cell membrane damage after exposure to 0, 10, 20, 40 and 80 μg/mL for 24 h. In addition, ZnONPs triggered ROS production, lipid peroxidation and activation of the apoptosis phenomenon [71]. Mitochondria damage was also observed in human dermal fibroblasts due to the activation of protein p53 cells after 4h of exposure to 10, 50, and 100 µg/mL of 20 nm ZnONPs [72]. Different shapes of AgNPs (rods 50 nm, spheres 50 nm and 20 nm triangles) were analyzed by Tak et al. with particular reference to their ability to induce skin permeation. In vitro analysis was carried out by the Franz diffusion cell system: the concentratin of silver rods, spheres and triangles decreased from 1.82, 1.17 and 0.52 μg/cm^2^ of skin after 12 h. In vivo studies on SKH-1 hairless mice showed higher skin penetration rates for rods than for spheres and triangles. Furthermore, they showed two ways to skin penetration: via follicoli and via intracellular spaces [73]. Rancan et al. studied the keratinocyte penetration of amorphous SiO_2_NPs with different surface charges (positive and negative) and sizes 291 ± 9 to 42 ± 3 nm. They showed that only smaller NPs were able to penetrate the damaged stratum cornum. For this reason, people with skin diseases such as dermatitis and chronic eczema can have several toxic effects due to SiO_2_NPs penetration [121]. A total of 20 nm and 100 nm SiO_2_NPs with positive and negative charge were used by Park et al. on keratinocytes. The results showed that smaller NPs promoted ROS production [74]. Nabeshi et al. showed a growth inhibition of HaCaT after exposure to 70 and 300 nm SiO_2_NPs in a size-dependent manner [75]. Sykes et al. observed a different distribution of 15 nm AuNPs in the skin of CD-1 nude athymic mice from topical or intravenous application (Figure 4). Only intravenous administration induced a flux of NPs in the blood vessels [78]. Pernodet et al. assessed the effects of AuNP citrate-capped on human dermal fibroblasts at different concentrations. The authors showed a destruction of actin stress fibers and a consequent viability reduction [76].

The reported data confirmed the high potential of NPs to penetrate the skin. However, this entry route is rather unlikely to be the favorite, as NPs will be able to cross the epidermis only through follicles or damaged skin.

## 4. Inorganic NPs Trigger Immune Response

**Figure 5:**
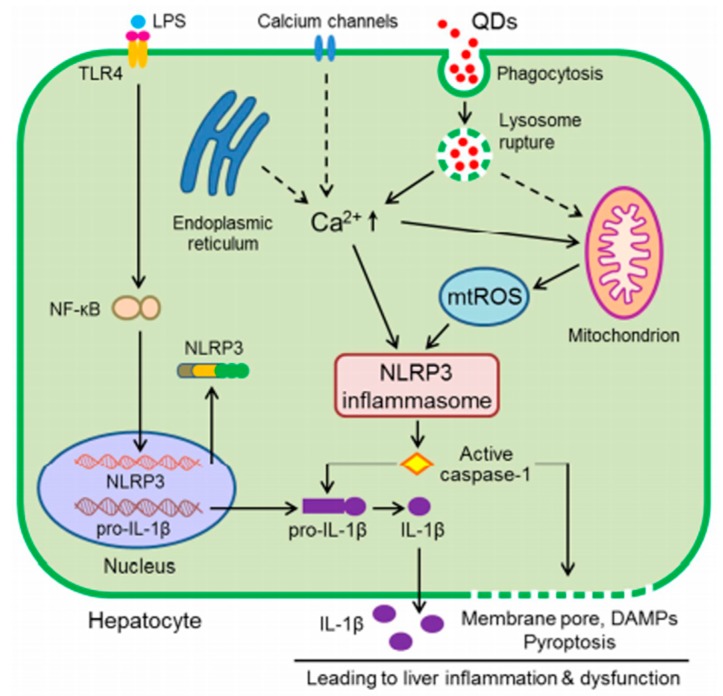
Mechanism of inflammation inducted by QDs. The uptake of QDs triggers a calcium flux inside cells and ROS production, which in turn induces NLRP3 infammasome activation. This phenomenon stimulates IL-1β maturation, which causes liver inflammation. Reprinted from reference [143]. Copyright (2016), with permission from Elsevier.

The immune system is vital for the living organism to maintain homeostasis due to its function to protect organisms from harmful agents. A lot of diseases are caused by the dysregulation of immune system [122,123]. The innate immune system includes physical barriers (mucus layers, cell–cell contacts, epitelia that cover the gastrointestinal and respiratory tracts) and molecules released by phagocytic cells, such as cytokines, chemokines and enzymes. Cytokines are crucial to determining the type of immune response. In particular, the cytokine interleukine-1β (IL-1β) is controlled by inflammasomes. Instead, the adaptive immune system has a strong specificity for the antigens; the receptors expressed on the lymphocytes T and B are at the basis of this immune response [124]. The information about the effects of NPs on the immune system is still limited. A lot of studies have been conducted on animal models or immortalized human cells in order to understand the interaction with the immune system, but the information is rather fragmented, and still needs to be deeply studied. The interaction between NPs and the immune system is influenced by the NPs’ physico-chemical properties such as size, shape, surface charge, stability and solubility, and crystalline forms [125,126,127]. Knowledge regarding immune responses is an important pillar to evaluate the cytotoxicity of NPs in order to design NMs to be used in nanomedicine for drug delivery, imaging and immune stimulation. Alveolar macrophages (AM) and neutrophils activated oxidative stress in the lungs after NP exposure. Phagocytic cells of the immune system, including neutrophils, induce ROS production through the activation of NADPH oxidase [128]. Several studies concluded that small and cationic NPs are more able to induce an inflammatory response than larger and anionic/neutral ones [129]. The positive surface charge on NPs attracts negatively-charged blood proteins. Proteins form a “protein corona” on the NP surface [130,131], inducing different effects on immune reactions as the cytokines release. Tsugita et al. investigated the effects of SiO_2_ and TiO_2_ on macrophage inflammatory responses. The NPs (~100 μg/head) were administrated into C57BL/6 N 6–7 week old female mice. After 24 h they showed IL-1β secretion in the macrophages that triggered lung inflammation. Alveolar macrophages have a crucial role in the pro- and anti-inflammatory response in alveoli, enabling the release of cytokines. TiO_2_NPs induced ROS production, whereas SiO_2_NPs stimulated lysosomal stress. This phenomenon activated the inflammasome due to macrophage induction [132]. Kongseng et al. reported an accurate study about the toxicity of TiO_2_NPs with a size of 29 ± 0.83 nm on human blood cells (peripheral blood mononuclear cells [PBMCs]). The exposure to NPs with dosages higher than 25 μg/mL for 24 h induced high secretion of IL-6 and tumor necrosis factor-α (TNF-α). In addition, several expressions of cyclooxygenase-2 (COX-2) and IL-1β were showed when cells were exposed to a concentration ≥125 μg mL^−1^ of TiO_2_NPs [133]. Murphy et al. investigated the effects of AgNPs coated with PVP (<100 nm) on the innate immune system. In particular, the authors evaluated the effects of AgNPs in a dose range between 1.9 to 250 µg/mL in THP-1 monocytes and primary blood monocytes on transcriptional expression of IL 1–6 and TNF-α. The up-regulation of IL-1, IL-6 and TNF-α caused by AgNPs underlined their potential inflammatory effects [134]. A total of 200 μg/mL of AgNPs, with a size of 5 nm, functionalized with tiopronin-induced IL-6 from macrophages [135]. A total of 4, 20, and 70 nm AgNPs were used to assess adverse effects in a human macrophage cell line (U937). After 24 h, the smaller NPs induced ROS generation and IL-8 secretion compared to larger NPs. These results suggested the important role of size in the inflammatory response [136]. Khan et al. studied the effects of kidney and liver exposure to uncoated AuNPs (10 and 50 nm) for a period of one to five days in rats, and they studied the modulation of IL-1β, IL-6, and TNF-α expression. The authors found an increase of cytokine gene expression induced by 50 nm AuNPs in the liver more than in the kidney [137]. Falagan-Lotscha et al. assessed the adverse effects of AuNPs (nanospheres and nanorods with different coatings) for up to 20 weeks on human dermal fibroblasts (HDFs). The nanospheres were functionalized with poly (acrylic acid) (PAA) or citrate and nanorods with PAA or polyethylene glycol (PEG). The nanorods induced several changes in gene expression; the expression of IL-6 was 12-fold with respect to control cells [138]. Durocher et al. [139] and Chen et al. showed the in vivo influence of AuNPs on the immunocompetent cells of mice; they found a high production of IL-2 and a proliferation of normal killers and lymphocytes [140]. In a recent work, Senapati et al., investigated the effects on the immune system of 20 nm ZnONPs in juvenile and adult BALB/c mice by sub-acute exposure. The results showed a modification in CD4 and CD8 cells. In adult mice, IL-6, IFN-γ, TNF-α and ROS were found to be increased, whereas in juvenile mice the same molecule levels did not show significant modification compared to the adults. Therefore, there was an increase of mitogen-activated protein kinase (MAPK) only in the aged mice, which suggested that ZnONPs have a strong ability to induce inflammatory response [141]. Giovanni et al. published an interesting work about the proinflammatory response of RAW264.7 macrophages, using ultra-low concentrations of AgNPs (35 nm), TiO_2_NPs (31 nm), and ZnONPs (32 nm). The three types of NPs were used at doses of 10 to 10^−7^ µg mL^−1^. Toxicity was observed when the cells were exposed to 10 µg mL^−1^, but the induction of NF-κB and the increase of expression of pro-inflammatory cytokines was observed at 10^−7^ µg mL^−1^ [142]. QDs also induced the alteration of elements of the immune system. Lu et al. showed the effects of CdSe/ZnS core/shell QDs in hepatic L02 cells. They observed a cytotoxicity that increases with the concentration of NMs (from 5 to 80 nM). In hepatocytes, QDs activated the NLR pyrin domain containing three inflammasomes (NLRP3), which started a process named pyroptosis, consisting of a pro-inflammatory form of cell death. Figure 5 shows the mechanism based on inflammation inducted by QDs [143].

Similar results were obtained by Wang et al., who conducted immunototoxicity experiments using CdSe/ZnS QDs on macrophages and lymphocytes. An increase of ROS production and apoptosis and a decrease of TNF-α and IL-6 was observed in macrophages incubated with 25 or 2.5 nM QDs. On the other hand, QDs induced high levels of release of TNF-α and IL-6 in lymphocytes [144].

## 5. Biodistribution and Toxicity of Inorganic NPs In Vivo after Intravenous Injection

Many in vitro studies showed the ability of inorganic NPs to induce DNA damage, induction oxidative stress, and apoptosis [145,146,147]. These observations were also supported by in vivo studies on rodents that investigated the toxicity of inorganic NPs administrated by different routes [148,149]. NPs translocated in organs (liver, kidneys, spleen, heart and brain) and tissues, by the blood system [150], in different ways that depend on their physico-chemical properties (size, shape, charge, surface coating, stability, crystallinity, agglomeration state and dosage). These parameters influenced the biokinetics and biological activity of NPs, including the translocation into organs from epithelia, intracellular localization, induction of ROS production and the connection to receptors [151]. The in vivo distribution of SiO_2_NPs with a size of 20.25 nm in nude mice was investigated. NPs were functionalized with a NIR dye and ^124^I for PET imaging. The authors found an accumulation in the liver and spleen corresponding to 70% of the total number of NPs, and about only 5% was in the kidney, heart and lung. SiO_2_NPs were excreted and did not show adverse effects in tissues [152]. Therefore, the surface properties influenced the accumulation of SiO_2_NPs in organs. He et al. used three different surface modifications of 45 nm SiO_2_NPS: OH-SiO_2_NPs (hydroxyl groups), COOH-SiO_2_NPs (carboxyl groups), and PEG-SiO_2_NPs (polyethylene glycol) following the distribution in mice through intravenous injection. They observed that OH-SiO_2_NPs and COOH-SiO_2_NPs were taken up more efficiently by the reticuloendothelial system than PEG-SiO_2_NPs. The latter also showed greater blood circulation times [153]. Borak et al. studied the excretion of SiO_2_NPs (150 nm) in mice investigating the in vivo distribution and toxicity of NPs administrated via injection in 100 μL of a series of doses (1, 2.5, 5, 10, 100, 200, and 300 mg/kg). Despite the distribution in many organs, the authors did not find definite toxic effects and they concluded that 36% of the NPs were eliminated by urine [154]. Previous works reported the biodistribution of AgNPs in organs such as the spleen, liver, lungs, and kidneys [155] after intravenous administration in mice [156,157,158] or rats [159,160]. Guo et al. assessed the in vivo toxicity of three different sizes of AgNPs and AgNO_3_ administered with single or multiple intravenous injections in female Balb/c mice (25 μg Ag for AgNPs and 2.5 μg Ag for AgNO_3_ per dose) for one, four and 10 days. The results showed the distribution and the toxicity in the liver, lungs and kidney that is caused by destruction of the endothelial barrier [161]. Nano-sized TiO_2_ (0, 324, 648, 972, 1296, 1944 or 2592 mg kg^−1^) was i.v. administered to mice, and the effects on serum at different times were observed. (24 h, 48 h, 7 days and 14 days). The examination of different tissues (lung, liver, kidney) showed a toxicity profile. The spleen presented lesions while the blood vessels were obstructed by the presence of TiO_2_NPs causing thrombosis. The high dosage of NPs also induced swelling of the renal glomerulus, hepatic fibrosis and liver necrosis [162]. A recent work by Jia et al. investigated the biodistribution and toxicity of micro-scale TiO_2_ (micro-TiO_2_) and 5, 10, 60, 90 nm of TiO_2_ in the anatase form. Different concentrations of NPs (5, 10, 50, 100, 150, and 200 mg/kg, once a day for 14 days) were administered to mice (22 ± 3 g, half male and half female) via intraperitonel injection. The authors found an accumulation in the brain, spleen, lungs and kidneys, which is proportional to the tested concentrations. The liver was strongly damaged due to mitochondrial disruption and the induction of apoptosis in hepatocytes. The smaller NPs were more toxic than the microsized NPs [163]. Takeuchi et al. [164] found a greater accumulation of 20 nm AuNPs in the lungs and brain after 2–3 h of administration in mice. In the pancreas, stomach and heart the route of accumulation is smaller and most of them were excreted. In fact, a value of 3–14.4% of AuNPs in feces and urine was observed. The biodistribution and toxicity of differently charged AuNCs were evaluated at a dose of 5.9 mg/kg After 1, 7, 30, 60, and 90 days of administration in 11-week-old male C57 mice. The negative AuNCs were more able to accumulate in the liver and spleen than neutral NPs. The peripheral blood system was partially damaged by positive AuNCs [165]. De Jong et al. followed the organ distribution in rats of AuNPs at different sizes (10, 50, 100, 250 nm). Upon i.v. administration, they observed a size-dependent toxicity and accumulation. The small NPs were able to accumulate in all the organs and the brain. In general, the blood, liver and spleen were the principal hosts of NPs [166]. Balasubramanian et al. studied a long-term collection of Au NPs (20 nm), showing a high level in rat spleens after two months [167]. The distribution of ZnO-NPs was analyzed after intraperitoneal injection (2.5 g/kg) in male Crl: CD-1 (ICR) mice (six-week-old). After 72 h, the ZnONPs were distributed in the liver, spleen and kidney. In particular, NPs triggered the progression of liver lesions. The in vitro evaluation conducted with an NP concentration of 20 mg/mL induced cytotoxicity and the production of a high level of oxidative stress elements [168]. Choi et al. observed a relation between the distribution of different amounts of zinc in organs and the blood and routes of administration. The rats treated with intravenous injection showed high levels of ZnONPs in the blood, whereas the oral administration (30 mg/kg) did not produce any relevant effect. The insufficient adsorption phenomenon by gastrointestinal tract was evident [169]. The blood clearance, distribution, excretion and toxicity of 8 µmol/kg body weight of Ag_2_Se QDs coated with PEG were investigated in mice by i.v. administration. QDs were translocated into circulation in 0.4 h and accumulated in the liver and spleen. The disaggregation into Ag and Se and their fate was investigated. Silver was eliminated more quickly by feces and urine than Se. However, a general toxicity of Ag_2_SeQDs was not detected experimentally [170]. Female BALB/c mice (10–12 weeks old) were exposed to fluorescent cadmium telluride/zinc sulfide (CdTe/ZnS) QDs that were subdermally injected with 20 μL QD2605 (20 μM). The QDs were taken up by the lymph nodes in four cell lines from different tissue sources [171].

## 6. Conclusions

Today, the business of NPs is growing in many fields. Their application ranges from medicine to cosmetics, as well as the construction, pharmaceutical and chemical industries. Despite their extraordinary properties, there are still “dark sides” related to their toxicity in living organisms. People are exposed to NPs through food and drinking water and skin products via the gastrointestinal and dermal penetration routes, respectively. Other relevant entry pathways are the respiratory system and the bloodstream. As described in this review, there are adverse effects of inorganic NPs due to their high accumulation in organs. This causes chronic toxicity over time due to the perpetual stimulation of the immune system that induces an inflammatory condition. In this state, a tissue may undergo malignant transformations. Moreover, some people may be more exposed to interaction with nanomaterials, especially those working on nanomaterial production. A lot of vivo and in vitro studies, together with computational predictive toxicity methods, are providing interesting data disclosing the specific mechanisms triggering the adverse effects of NMs. In addition, several research efforts are aimed at enabling the surface modification of NPs via a chemical approach, in order to make them safer and less toxic. Moreover, a crucial point is to establish a dosage at which a nanomaterial can be considered safe and thus suitable for daily consumer products: this goal can be achieved by standardized in vitro procedures to establish the toxicology profile of NPs [106]. This can be developed by the implementation of specific standard operating procedures (SOPs) for the testing of NPs. These procedures, based on principles of good laboratory practice (GLP), should be supported by researchers and scientists in order to analyze high amounts of data [172]. Future challenges will involve the creation of flexible and reliable databases in which NPs can be classified according to the results deriving from toxicological investigations. The different types of NPs should be listed indicating the safe concentrations, the type of toxicity, but also the specific protective device required according to the routes of entry into the body. In this way, the scientific research could support clearer and more certain legislation on the use of nanomaterials.

## Figures and Tables

**Table 1:** Principal studies concerning the route of entry of inorganic NPs in vitro and in vivo.

Exposure Routes	In Vitro	In Vivo
Ingestion	[37,38,39,40,41,42,43,44,45,46,47]	[48,49,50,51,52]
Ihalation	[53,54,55,56,57,58,59,60,61]	[62,63,64,65,66,67]
Skin penetration	[68,69,70,71,72,73,74,75,76,77]	[73,78]
